# A Case of Primary Renal Carcinoid Tumor

**DOI:** 10.1155/2015/736213

**Published:** 2015-01-22

**Authors:** Toshikazu Tanaka, Hayato Yamamoto, Atsushi Imai, Hatakeyama Shingo, Takahiro Yoneyama, Takuya Koie, Yasuhiro Hashimoto, Chikara Ohyama

**Affiliations:** Department of Urology, Hirosaki University Graduate School of Medicine, 5 Zaifucho, Hirosaki 036-8562, Japan

## Abstract

Primary renal carcinoid tumors are extremely rare kidney lesions, with fewer than 100 reported cases previously. We describe a 75-year-old man with an incidentally detected cystic renal mass. Computed tomography showed a 3 cm tumor with a cystic component enhanced with contrast. No evidence of metastasis was detected. We treated the patient with radical nephrectomy. Pathological examinations revealed a cellular arrangement specific to carcinoid tumor and positive for chromogranin A, neural cell adhesion molecule, and somatostatin receptor type 2. The tumor cells had a mitotic count of 4 mitoses/10 high-power fields, and the level of the proliferation marker Ki-67 was 5%. The pathological diagnosis was renal neuroendocrine tumor grade 2. No local recurrence and no systemic metastasis were detected during the 18-month follow-up period. To our knowledge, this is the 6th case of renal neuroendocrine grade 2 tumor reported thus far.

## 1. Introduction

Carcinoid tumors can arise in almost any organ, but they occur most commonly in the gastrointestinal and bronchopulmonary systems and less frequently in the hepatobiliary system [1, 2]. A primary renal carcinoid tumor is extremely rare, with only 97 cases reported in the medical literature [2–9]. We describe a 75-year-old man with a primary renal carcinoid tumor without any evidence of metastasis or carcinoid syndrome.

## 2. Case Presentation

**Figure 1:**
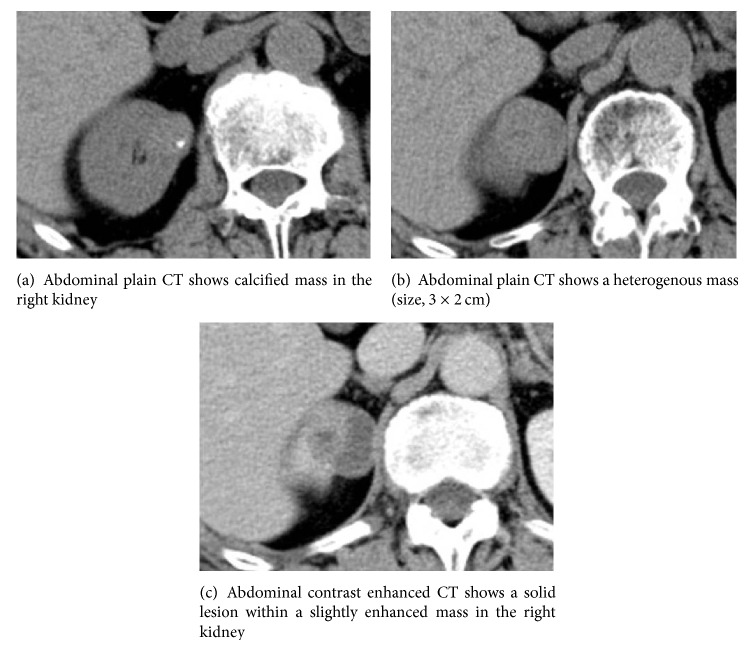
Abdominal computed tomography (CT).

**Figure 2:**
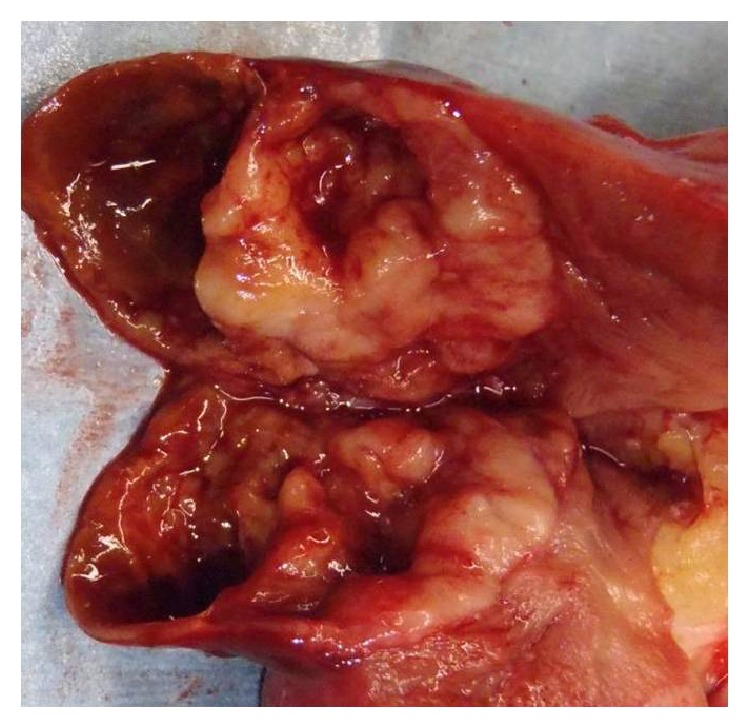
Macroscopic findings. The gross features of the resected tumor included a white-yellow soft mass attached to a cystic lesion.

**Figure 3:**
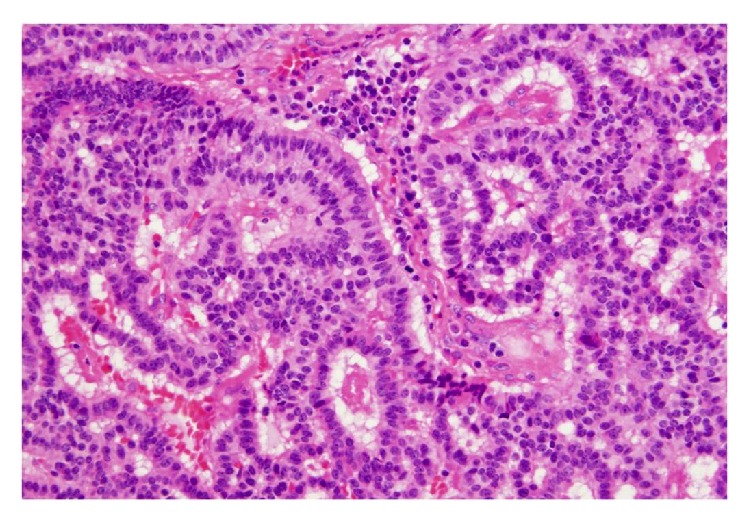
Microscopic findings. Tumor cells are arranged in cords and with a ribbon-like appearance (hematoxylin and eosin, magnification: ×400).

**Figure 4:**
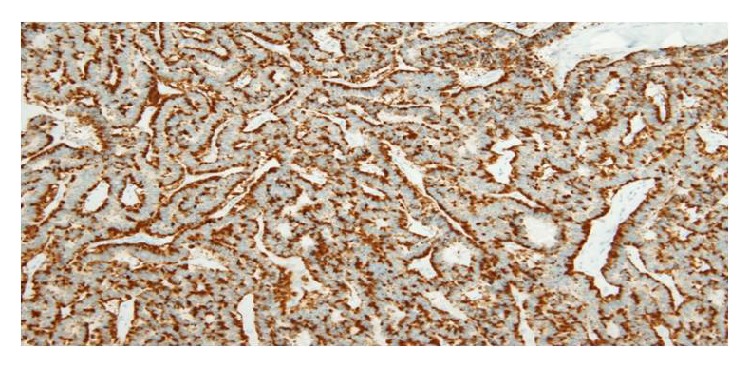
Chromogranin A-stained section. Tumor cells are positive for chromogranin A immunostain (magnification: ×200).

**Figure 5:**
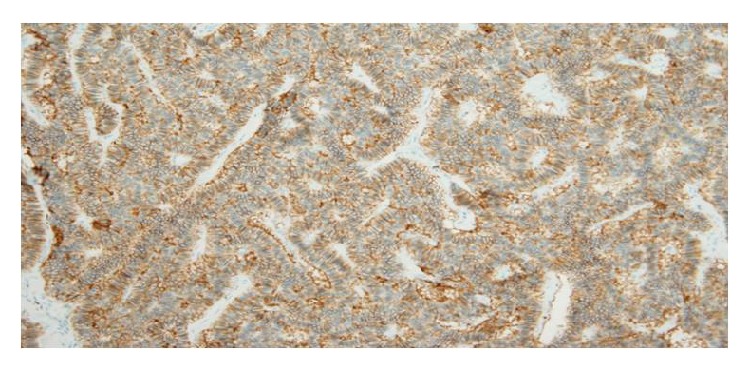
Neural cell adhesion molecule-stained section. Tumor cells are positive for neural cell adhesion molecule immunostain (magnification: ×200).

**Figure 6:**
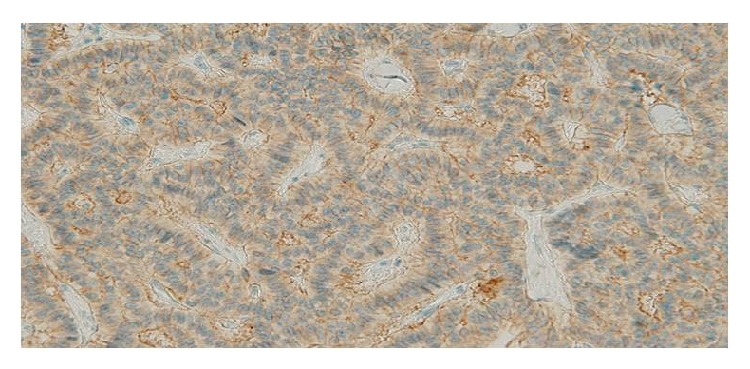
Somatostatin receptor type 2-stained section. Tumor cells are positive for somatostatin receptor type 2 immunostain (magnification: ×400).

A 75-year-old man was referred to our institution after being diagnosed incidentally with a 3.0 cm, solid, right renal mass on computed tomography (CT) performed to assess thoracic trauma. He had no specific symptoms. The results of laboratory investigations were within normal limits. On abdominal CT, a renal mass was identified in the upper portion of the right kidney (Figure 1). The mass measuring approximately 3.0 cm × 2.0 cm was well defined, and it consisted of a partial cystic mass within a mainly solid renal mass. The cystic component contained calcifications. No definite hydronephrosis or caliectasis was detected. The patient had no other abnormal findings, such as suspected distant metastasis or lymph node metastasis. At that time, we considered cystic renal cell carcinoma (RCC), oncocytoma, and adult-type cystic nephroma as the differential diagnosis of this mixed cystic and solid renal tumor. In May 2012, we planned an open partial right nephrectomy; however, during the operation, we noticed that the tumor had invaded the renal capsule. Therefore, we performed open right nephrectomy. After histopathological evaluation, the lesion was diagnosed as a primary renal carcinoid tumor (neuroendocrine tumor). The tumor met both the histological and immunochemical criteria for designation as a carcinoid tumor. Grossly, the resected tumor included a white-yellow soft mass attached to a cystic lesion (Figure 2). On microscopic examination, the tumor consisted of a ribbon-like, trabecular, or rosette like pattern of cells with a high nucleus: cytoplasm ratio (Figure 3). Immunohistologically, the tumor cells were positive for antibodies for the neuroendocrine markers, chromogranin A (Figure 4), neural cell adhesion molecule (NCAM) (Figure 5), and somatostatin receptor type 2 (SSTR2) (Figure 6). The tumor cells had a mitotic count of 4 mitoses/10 high-power fields and the level of proliferation marker Ki-67 was 5%. The microscopic and immunohistological findings were compatible with the diagnosis of a primary renal neuroendocrine tumor (grade 2). No local recurrence or systemic metastasis was detected during the 18-month follow-up period.

## 3. Discussion

Carcinoid tumors are low-grade malignant neoplasms with neuroendocrine differentiation. They are located mainly in the gastrointestinal (74%) and respiratory (25%) tracts [1]. In <1% of all cases, carcinoid tumors are reported in the testes, ovaries, kidneys, and the prostate [2]. A primary renal carcinoid tumor is extremely rare, with only 97 cases reported in the medical literature. Primary renal carcinoid tumors are equally prevalent in both genders [3]. Such tumors usually occur in patients aged between 13 and 79 years [4], with a mean age of occurrence lower than that for renal cell carcinoma [5].

Intrinsic neuroendocrine cells in normal kidneys have not been reported previously, and the pathogenesis of renal carcinoid tumors is controversial. Several theories regarding the origins of carcinoid tumors have been postulated. Neuroendocrine cells could be derived from neural crests or pancreatic tissue in the kidney during embryogenesis [6]. Carcinoid tumors in the kidney have been reported to arise most commonly in the setting of renal congenital and acquired anomalies such as horseshoe kidney and mature teratoma [7, 8]. It has been postulated that chronic inflammation induces intestinal metaplasia of the pelvicalyceal urothelium, which subsequently triggers derivation of interspersed neuroendocrine cells [4].

The most commonly reported presenting complaint in cases of carcinoid tumors is abdominal pain, and evidence of carcinoid syndrome with serotonin-related flushing, edema, and diarrhea is seen in less than 10% of cases [9]. The diagnosis of primary renal carcinoid tumor is incidental in 25–30% of cases [2]. Our patient did not present any symptoms, and he was diagnosed incidentally.

The radiographic findings of carcinoid tumors commonly indicate a solid tumor or a cystic component as well, and the presence of calcification has been reported in 26.5% of cases [10]. The tumors have heterogeneity and minimal enhancement in CT findings [11]. In our case, we observed similar findings on CT, such as calcification, solid component, and minimal enhancement. Octreotide scintigraphy and 68Ga-labeled positron emission tomography- (PET-) CT are useful tools for diagnosis, staging, and monitoring after treatment for the development of recurrence or metastasis of carcinoid tumors [12, 13]. Radiolabeled octreotide is a somatostatin analogue that has a high affinity for somatostatin receptors. Primary carcinoids and metastatic lesions do have high affinity receptors for somatostatin in 87% of cases [7].

Renal carcinoid tumors have the characteristic features of carcinoid tumors located elsewhere. The World Health Organization (WHO) has proposed a classification system for renal carcinoid tumors in different locations [14]. A classification system established in 2000 and updated in 2010 differentiates neuroendocrine tumors (NET) and neuroendocrine carcinomas [15]. Important factors in this classification are the proliferation index (Ki-67, MIB-1), angioinvasion, and mitoses. Tumors are divided into NET grade 1 (mitotic activity <2/10 high-power fields and ≤2% Ki-67 index), NET grade 2 (mitotic activity 2–20/10 high-power fields and/or 3–20% Ki-67 index level), and neuroendocrine carcinoma (mitotic activity >20/10 high-power fields and >20% Ki-67 index level). The patient described herein had a mitotic activity of 4/10 high-power fields and 5% Ki-67 index level. Thus, the tumor was classified as NET grade 2. NET grade 2 tumors are rare, with only one reported case in a large study by Hansel et al. [8]. Further, we have been able to identify only five cases of NET grade 2 tumors reported in the literature [8, 16–18].

The most predominant histological pattern of carcinoid tumors is the trabecular or ribbon-like pattern [8]. Immunohistochemical stains were consistent with the diagnosis of carcinoid tumor, with the majority of the cases presenting strong immunoreactivity for synaptophysin, chromogranin A, NCAM, and somatostatin receptor type 2 [2, 19]. In our case, the tumor cells were positive for antibodies against the neuroendocrine markers chromogranin A, NCAM, and somatostatin receptor type 2.

The current recommended management for localized primary renal carcinoid tumors includes surgical resection of the kidney, which may be open radical or partial nephrectomy with lymph node dissection. In some cases, metastatic renal carcinoid tumors are resistant to chemotherapy [9]. Recently several treatments for carcinoid tumors have been reported, such as somatostatin analogues, everolimus, and radionuclide therapy [20–22]. Adjuvant therapy is not currently indicated in patients with completely resected NETs [23]. But the results of a placebo-controlled randomized double blind prospective phase IIIB study on the effect of octreotide on tumour growth in patients with metastatic midgut NETS (PROMID Study) showed that octreotide significantly lengthens time to tumour progression (14.3 months in octreotide group versus 6 months in the placebo group) in patients with functionally active and inactive NETs [20]. Conventional methods of imaging are inadequate for detecting smaller carcinoids, so octreotide scintigraphy or 68Ga-labeled PET-CT should complement computed tomography and magnetic resonance imaging when searching for occult or metastatic disease postoperatively. And we should consider whether to adjuvant therapy based on the nuclear examination.

The prognosis of renal carcinoid tumors is not predictable because of their rarity. Omiyale and Venyo found metastasis in 55% of the 29 patients under study and reported that 73.1% of the 29 patients did not present evidence of disease after treatment [3]. Although prognosis of renal carcinoid tumors is better than that for other renal neoplasms, renal NETs grade 2 are associated with poor prognosis. All of the five renal NET grade 2 cases previously reported had metastasis and 2 of 5 (40%) patients died because of the disease [8, 16–18]. In our case, no local recurrence and no systemic metastasis were detected in the 18-month follow-up period. However, we did not perform any nuclear medicine images for searching occult or metastatic disease. A nuclear medicine image and strict follow-up after surgery were needed because of the poor prognosis associated with renal NET grade 2 tumors.
